# Immunogenicity of BNT162b2 Vaccine Booster Dose in Patients With Inflammatory Bowel Disease Receiving Infliximab Combination Therapy: A Prospective Observational Study

**DOI:** 10.3389/fmed.2022.933996

**Published:** 2022-07-04

**Authors:** Mohammad Shehab, Fatema Alrashed, Ahmad Alfadhli, Abdulwahab Alsayegh, Usama Aldallal, Mariam Alsayegh, Preethi Cherian, Irina Alkhair, Thangavel Alphonse Thanaraj, Arshad Channanath, Ali A. Dashti, Anwar Albanaw, Hamad Ali, Mohamed Abu-Farha, Jehad Abubaker, Fahd Al-Mulla

**Affiliations:** ^1^Division of Gastroenterology, Department of Internal Medicine, Mubarak Alkabeer University Hospital, Kuwait University, Kuwait City, Kuwait; ^2^Department of Pharmacy Practice, Faculty of Pharmacy, Health Sciences Center (HSC), Kuwait University, Kuwait City, Kuwait; ^3^Department of Internal Medicine, School of Medicine, Royal College of Surgeons in Ireland, Medical University of Bahrain, Busaiteen, Bahrain; ^4^Department of Biochemistry and Molecular Biology, Dasman Diabetes Institute (DDI), Dasman, Kuwait; ^5^Department of Genetics and Bioinformatics, Dasman Diabetes Institute (DDI), Kuwait City, Kuwait; ^6^Department of Medical Laboratory Sciences, Faculty of Allied Health Sciences, Health Sciences Center (HSC), Kuwait University, Kuwait City, Kuwait

**Keywords:** IBD, vaccine, infliximab, COVID-19, immunogenicity, booster

## Abstract

**Introduction:**

Few data exist regarding the immunogenicity of the third dose of BNT162b2 relative to the second dose in patients with inflammatory bowel disease (IBD) on different immunosuppressive therapies. We investigated the immunogenicity of BNT162b2 vaccine booster dose in patients with IBD on infliximab combination therapy.

**Method:**

This is a prospective single-center observational study conducted from January 1, 2022 to February 28, 2022. Patients were recruited at the time of attendance at the infusion center. Eligibility criteria included patients with a confirmed diagnosis of IBD who are receiving infliximab with azathioprine or 6-mercaptopurine. Patients who received two doses of BNT162b2 vaccine (second dose group) were compared to patients who had received three doses of BNT162b2 vaccine [third dose (booster) group]. Patients were excluded if they were infected or had symptoms of severe acute respiratory syndrome coronavirus 2 (SARS-CoV-2) previously since the start of the pandemic or received other vaccines than the BNT162b2. Our primary outcome was the concentrations of SARS-CoV-2 antibodies Immunoglobulin G (IgG) and neutralizing antibodies 40–45 weeks from the first dose of BNT162b2 vaccine in patients with IBD receiving infliximab combination therapy. Medians with interquartile range (IQR) were calculated.

**Results:**

In total, 162 patients with IBD and receiving infliximab combination therapy were recruited, and the number of patients in both the second dose group and third dose (booster) group was 81. Mean age was 35 years old in both groups. Median (IQR) SARS-CoV-2 IgG levels were significantly lower after the second dose [125 BAU/ml (43, 192)] compared to patients who received the third booster dose [207 BAU/ml (181, 234)] (*P* = 0.003). Neutralizing antibody levels were also lower after the second dose [80% (21, 95)] compared to patients who received the third booster dose [96% (93, 99)] (*P* ≤ 0.001). The percentage of patients who achieved positive SARS-CoV-2 IgG levels in the third (booster) dose group was 96.3%, whereas it was 86.4% in the second dose group. The percentage of participants who received the third (booster) dose and achieved a positive SARS-CoV-2-neutralizing antibody level was 100%, whereas it was 88.9% in the participants who received the second dose only.

**Conclusion:**

Most patients with IBD on infliximab combination therapy had positive SARS-CoV-2 IgG and neutralizing antibody concentrations 40–45 weeks post BNT162b2 vaccination. However, SARS-CoV-2 IgG and neutralizing antibody concentrations were lower in patients who received two doses only compared to patients who received a third dose. A longer follow-up study is needed to evaluate decay in antibodies over time.

## Introduction

Coronavirus was first identified to have infected people in the mid-1960s. Since then, multiple subgroupings have emerged. Recent and most notable of which have been severe acute respiratory syndrome coronavirus 2 (SARS-CoV-2) originating from Wuhan, China (1, 2). SARS-CoV-2 is particularly dangerous in part due to its heterogeneous symptoms resulting in anything between diarrhea to severe respiratory distress and its high infectious rate. The transmission of the virus occurs *via* the presence of angiotensin-converting enzyme 2 (ACE2) receptors, present on the epithelial type II cells in the lungs and the brush border of gut enterocytes. Here, the virus can then utilize these receptors to access the host's tissue resulting in infection (3). As a result of its rapid spread, SARS-CoV-2, as of May 23, 2022, has infected 525,467,084 confirmed cases, of which 6,285,171 resulted in deaths (4). Despite new variants like Delta and Omicron continuing to cause a global surge in cases, the advent of vaccines, such as boosters, brings confidence that the spread will be curbed.

Global efforts led to the development of highly effective COVID-19 vaccines, with early findings reporting 95% efficacy of mRNA vaccine BNT162b2 against COVID-19 (5, 6). Since then; however, variants sprouted and immunity dwindled. This resulted in the reported efficacy of BNT162b2 against Omicron dropping to 34–37% after 15 weeks of the second dose. Thankfully, the arrival of boosters for BNT162b2 primary course recipients helped push COVID-19 vaccine efficacy back over 70%, making it especially important for the immunocompromised patients such as those with inflammatory bowel disease (IBD) (7).

Inflammatory bowel disease is a chronic immune-mediated inflammatory process often distinguished between two subtypes, Crohn's disease (CD) and Ulcerative Colitis (UC), impacting millions of patients. IBD is often treated using immunosuppressive drugs like corticosteroids, tumor necrosis factor (TNF) inhibitors, thiopurines, and Janus kinase inhibitors, raising concerns over the risk of patient complications from infectious sources. This is exceptionally significant today, as recent evidence suggests that corticosteroid and 5-aminosalicylic acid (5-ASA) use is associated with more severe COVID-19 outcomes such as hospitalization and death (8–11). This can be explained due to a blockage of intracellular signals needed for the host to fight pathogens with patients on immunosuppressive drugs (12, 13). Furthermore, early findings in patients receiving biological treatments such as infliximab combination therapy demonstrated lower SARS-CoV-2 IgG, IgA, and neutralizing antibody levels after BNT162b2 vaccination compared with healthy participants (14). In addition, a previous study demonstrated that the immunogenicity of COVID-19 vaccines varies according to the immunosuppressive drug exposure and is attenuated in recipients of infliximab, infliximab plus thiopurines, and tofacitinib (15). All things considered, most patients displayed significant immunogenicity after two doses of the vaccines, illustrating the potential greater benefits of a third dose (16). Thus, this study aims to assess the immunogenicity of the second dose compared to the third (booster) dose of BNT162b2 vaccine in patients with IBD receiving infliximab with azathioprine or 6-mercaptopurine (infliximab combination).

## Materials and Methods

A prospective single-center observational study was conducted at a tertiary care IBD center, Mubarak Al-Kabeer Hospital. Patients were recruited at the time of attendance at the infusion center from January 1, 2022, to February 28, 2022. Patients who received two doses of BNT162b2 vaccine (second dose group) were compared to patients who had received three doses of BNT162b2 vaccine [third dose (booster) group]. This study was performed and reported in accordance with Strengthening the Reporting of Observational Studies in Epidemiology (STROBE) guidelines (17). The international classification of diseases (ICD-10 version: 2016) was used to diagnose IBD. Patients were considered to have IBD when they had ICD-10 K50, K50.1, K50.8, K50.9 corresponding to CD and ICD-10 K51, k51.0, k51.2, k51.3, k51.5, k51.8, k51.9 corresponding to UC (18).

**Figure 1:**
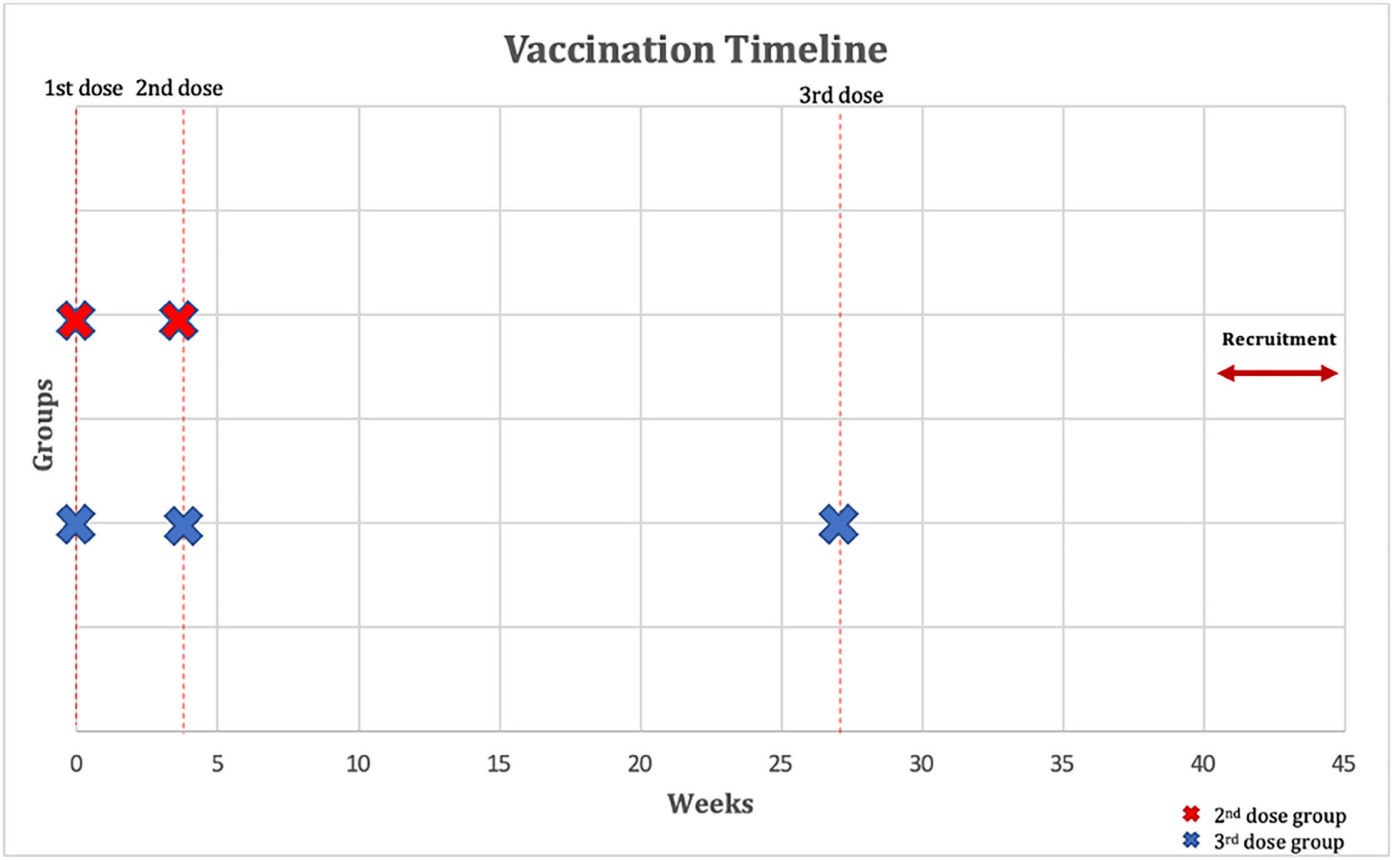
Outline of vaccination timeline and time when a serological response was assessed. The figure shows that participants have received two-dose of COVID-19 vaccination with BNT162b2 vaccine 3 weeks apart and that the third (booster) dose group have received a third dose with BNT162b2 vaccine 24 weeks after the second dose. Immunoglobulin G (IgG) and neutralizing antibodies were measured 40–45 weeks after their first dose of BNT162b2.

Patients were eligible to be included if they: (1) had a confirmed diagnosis of IBD before the start of the study; (2) were receiving infliximab with azathioprine or 6-mercaptopurine for at least 6 weeks or more for the induction of remission or with at least one dose of drug received for the maintenance of remission in the previous 8 weeks before the first dose of vaccination; (3) Second dose group: have received two-dose of COVID-19 vaccination with BNT162b2 vaccine, 3 weeks apart. Third dose (booster) group: have received two-dose of COVID-19 vaccination with BNT162b2 vaccine, 3 weeks apart and a third (booster) dose 24 weeks after the second dose (Figure 1); (4) were at least 18 years of age or older. SARS-CoV-2 PCR was performed within 72 h of each vaccine dose, and if positive, patients were excluded. Patients were also excluded if they were infected or had symptoms of SARS-CoV-2 previously since the start of the pandemic. In addition, patients who received other vaccines than the BNT162b2 were excluded. Patients who received corticosteroids 2 weeks before the first dose of the vaccine up to the time of recruitment were also excluded. Finally, patients taking other immunomodulators such as methotrexate were also excluded.

### Outcome Measures

Our primary outcome was the concentrations of SARS-CoV-2 antibodies, including Immunoglobulin G (IgG) and neutralizing antibodies 40–45 weeks after their first dose of BNT162b2 in patients with IBD receiving infliximab combination therapy. Data regarding the type and extent of IBD as well as duration of infliximab combination therapy were also recorded.

### Laboratory Methods

Enzyme-linked immunosorbent assay (ELISA) kit (SERION ELISA agile SARS-CoV-2 IgG and IgA SERION Diagnostics, Würzburg, Germany) was used to measure plasma levels of SARS-CoV-2-specific IgG antibodies based on the manufacturer's protocol. Units of IgG levels were reported as binding antibody units (BAU)/ml. Values below 31.5 BAU/ml were considered negative or non-protective.

The SARS-CoV-2-Specific Neutralizing Antibodies levels in the plasma were quantified using a SARS-CoV-2-specific surrogate Virus Neutralization Test (sVNT) (SARS-CoV-2 sVNT kit, GenScript, USA, Inc.) based on the manufacturer's protocol. Determination of positive and negative thresholds was performed by following the manufacturer's recommendations. The results were interpreted by calculating inhibition rates for samples per the following equation: Inhibition = (1–sample O.D/O.D negative control) ×100%. Neutralizing antibody levels below 20% were considered negative or non-protective.

### Ethical Consideration

This study was reviewed and approved by the Ethical Review Board of Dasman Institute “Protocol # RA HM-2021-008” as per the updated guidelines of the Declaration of Helsinki (64th WMA General Assembly, Fortaleza, Brazil, October 2013) and of the US Federal Policy for the Protection of Human Subjects. The study was also approved by the Ministry of Health of Kuwait (reference: 3799, protocol number 1729/2021). Subsequently, patient informed written consent was obtained before inclusion in the study.

### Statistics

We performed descriptive statistics to characterize both the second and third (booster) dose groups. The standard descriptive statistics were used to present the demographic characteristics of patients included in this study and their measured antibody levels. Analysis was conducted in R (19). Data are expressed as medians with interquartile range (IQR) unless otherwise indicated. Categorical variables were compared using the Fisher's exact test or Pearson's chi-squared test. The unpaired and paired continuous variables were compared with the Wilcoxon rank-sum test and Wilcoxon signed-rank exact test, respectively. A *P*-value of ≤ 0.05 is considered statistically significant.

Both groups were matched for age, sex, and time-since-first vaccine-dose using the optimal pair matching method. The technique attempts to choose matches that collectively optimize an overall criterion. The criterion used was the sum of the absolute pair distances in the matched sample. In addition, the percentage of positive IgG and neutralizing antibody levels was calculated in both groups. The χ^2^ tests were used to assess whether the percentages of positive antibodies differed across categories of both groups.

## Results

### Baseline Cohort Characteristics

Patients were recruited between January 1, 2022 and February 28, 2022. In total, 162 patients were recruited, and serology assays were performed to quantify SARS-CoV-2 antibody levels for all patients. The number of patients included in both the second dose group and third (booster) dose group was 81. The mean age was 35 years old in both groups, and body mass index (BMI) was lower in the second dose group compared to the third dose group (25.8 vs. 24.7 kg/m^2^). In both groups, most patients had CD (>56%), and more than 20% of patients were smokers. The mean duration between the serology test and second dose of vaccine was 40 weeks, and the mean duration between the serology test and third (booster dose) was 16 weeks. The median duration of infliximab combination therapy was 12 months (see Table 1).

**Table 1:** Baseline characteristics of participants.

**Characteristic**	**Second dose, *N* = 81**	**Booster dose, *N* = 81**
Mean Age (years)	35.2	35.6
**Gender** ***n*** **(%)**		
Female	38 (47%)	36 (45%)
Male	43 (53%)	45 (55%)
BMI (median)	24.7	25.8
Smoking *n* (%)	19 (23.0%)	17 (21.0%)
**Co-morbidities** ***n*** **(%)**		
Diabetes	3 (3.7%)	3 (3.6%)
Hypertension	7 (8.6%)	6 (7.1%)
Heart disease	5 (6.1%)	6 (7.1%)
Arthritis or any autoimmune disease	8 (9.8%)	9 (11%)
COPD	1 (1.2%)	1 (1.2%)
Kidney disease	2 (2.4%)	0 (0%)
Asthma	2 (2.5%)	1 (3.6%)
Hyperlipidemia	9 (11%)	9 (11%)
Duration of infliximab combination therapy (median, months)	12	13
**Disease extent** ***n*** **(%)**		
Ulcerative colitis (UC)	33 (41%)	36 (44%)
E1: ulcerative proctitis	5 (16%)	6 (18%)
E2: left sided colitis	11 (32%)	12 (33%)
E3: extensive colitis	17 (52%)	18 (49%)
Crohn's disease (CD)	48 (59%)	45 (56%)
L1: ileal	26 (54%)	23 (51%)
L2: colonic	5 (10%)	5 (11%)
L3: ileocolonic	17 (36%)	15 (34%)
L4: upper gastrointestinal	0 (0%)	2 (4%)
B1: inflammatory	21 (44%)	19 (43%)
B2: stricturing	13 (28%)	12 (27%)
B3: penetrating	14 (30%)	14 (30%)
**Lab parameters**		
CRP, mg/L (median)	6.3	6.2
Albumin, g/L (median)	40	42
ESR, mm/h	11	9
Stool fecal calprotectin, μg/g (median)	114	112

### Outcomes

**Figure 2:**
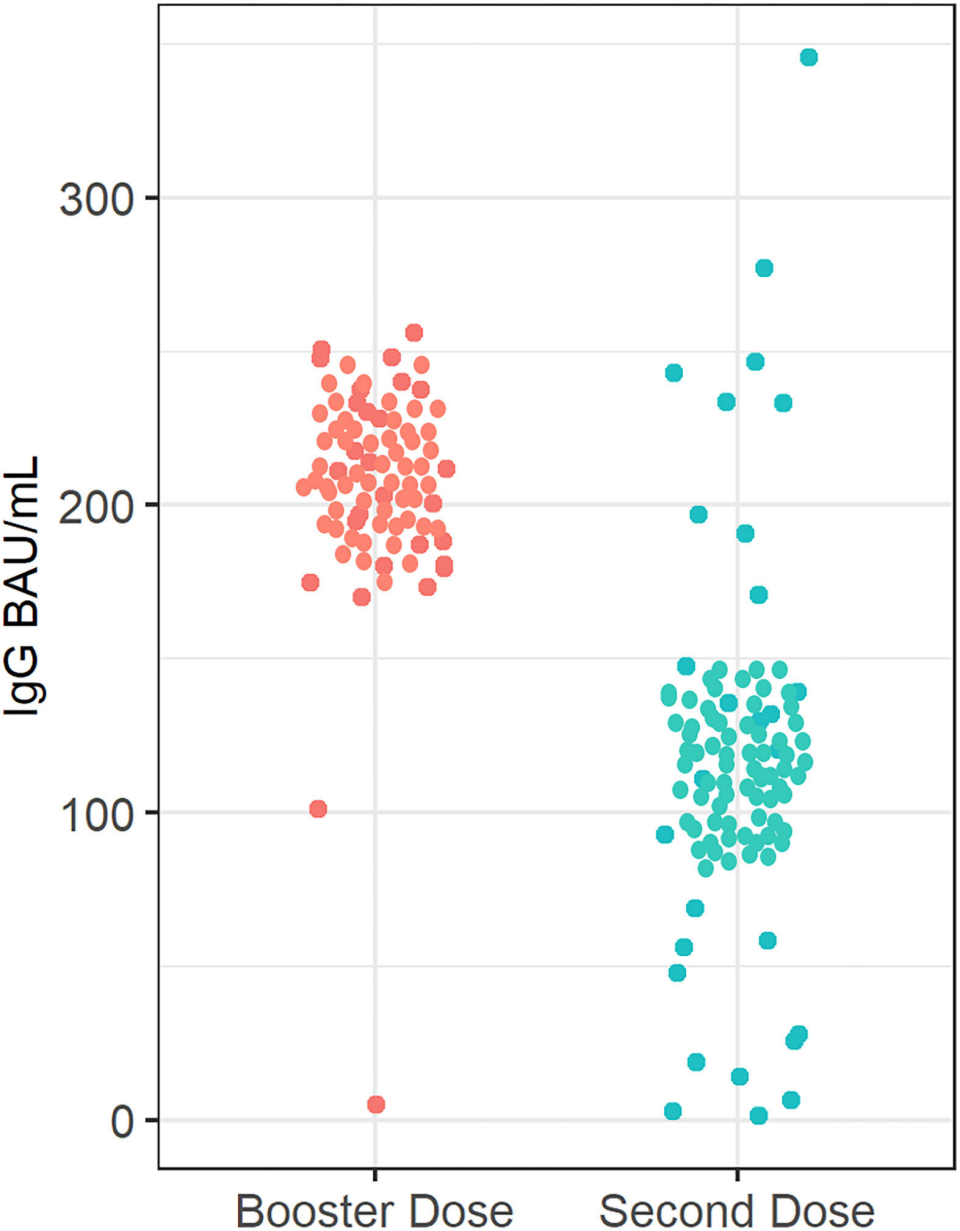
Anti-SARS-CoV-2 -IgG antibody concentrations in patients receiving infliximab combination therapy after the second and third (booster) dose. The median anti-SARS-CoV-2 IgG concentration after receiving the second dose was 125 BAU/ml, whereas the median anti-SARS-CoV-2 -IgG antibody concentration was 207 BAU/ml after receiving the third (booster) dose.

**Figure 3:**
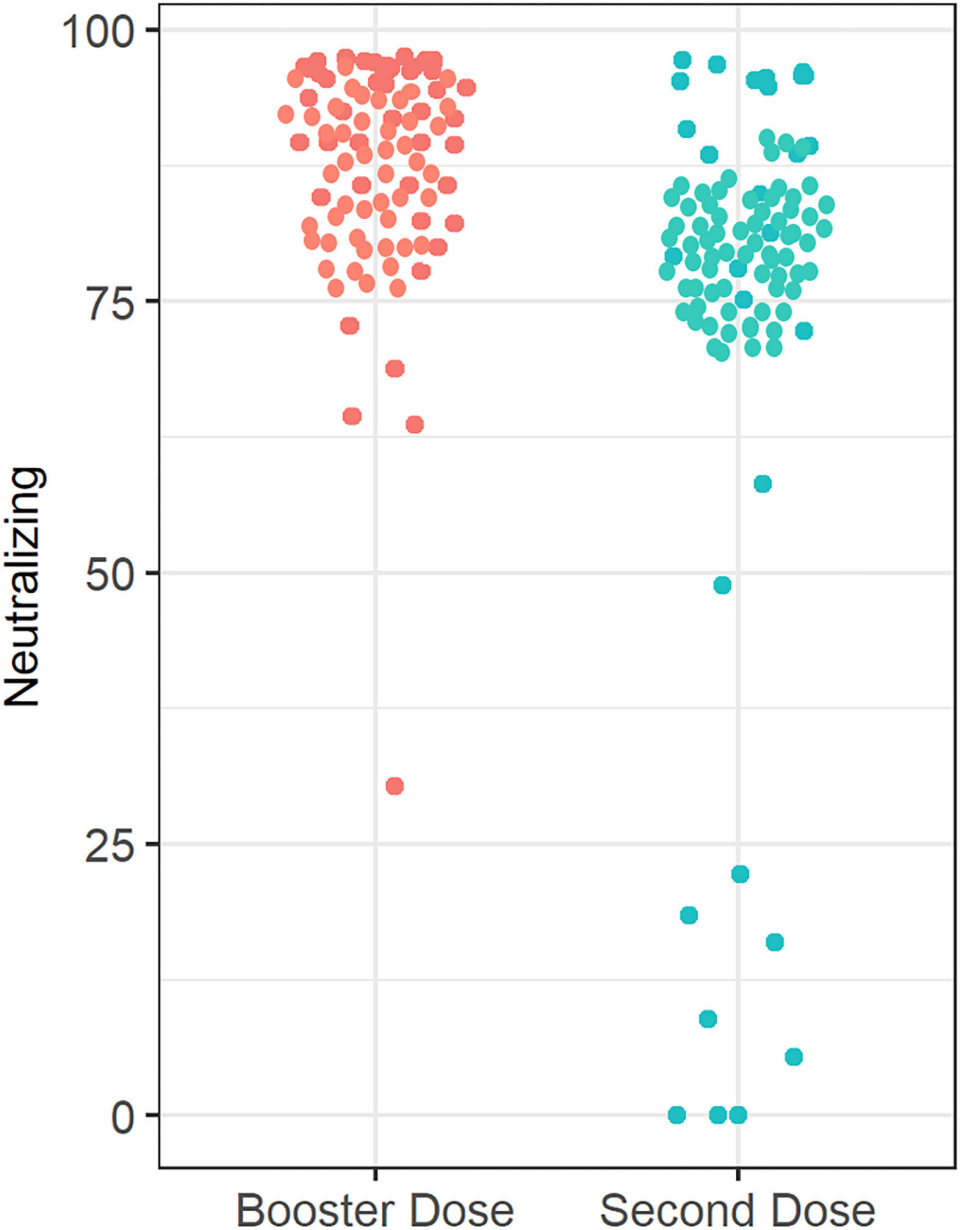
Anti-SARS-CoV-2 neutralizing antibody concentrations in patients receiving infliximab combination therapy after the second and third (booster) dose. The median anti-SARS-CoV-2-neutralizing antibody concentration after receiving the second dose is 80%, whereas the median neutralizing antibody concentration was 96% after receiving the third (booster) dose.

Median (IQR) SARS-CoV-2 IgG level was significantly lower in patients treated with infliximab combination therapy after the second dose [125 BAU/ml (43, 192)] compared to patients who received the third booster dose [207 BAU/ml (181, 234)] following vaccination with BNT162b2 (*P* = 0.003) (see Figure 2). Neutralizing antibody levels were also lower in patients who were treated with infliximab combination therapy after the second dose [80% (21, 95)] compared to patients who received the third booster dose [96% (93, 99)] following vaccination with BNT162b2 (*P* ≤ 0.001) (see Table 2 and Figure 3).

**Table 2:** Antibody responses in patients receiving infliximab combination therapy after third (booster) dose vs. second dose.

**Type of antibody test**	**Second dose, *N* = 81** ***^a^***	**Booster dose, *N* = 81** ***^a^***	***P*** **-value** ***^b^***
IgG BAU/ml	125 (43, 192)	207 (181, 234)	0.003
Neutralizing antibody (%)	80 (21, 95)	96 (93, 99)	<0.001

a *Median (IQR)*.

b *Wilcoxon signed-rank exact test; random intercept logistic regression*.

**Figure 4:**
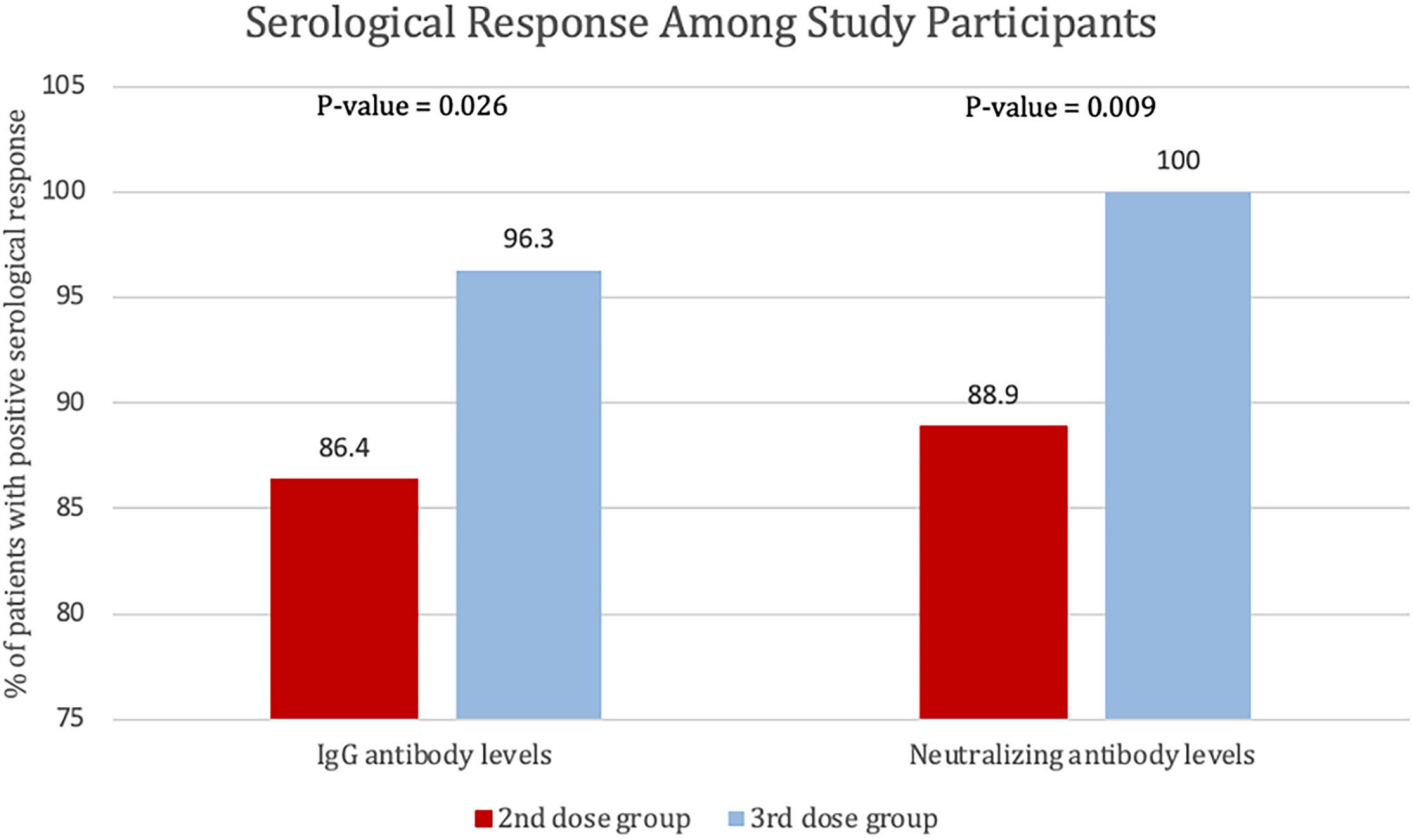
Percentage of patients with positive serological response to the second and third dose of BNT162b2, defined by SARS-CoV-2 IgG antibody levels of >31.5 BAU/ml or SARS-CoV-2-neutralizing antibody levels >20%.

The percentage of patients who achieved positive SARS-CoV-2 IgG levels in patients who received the third (booster) dose was 96.3% (78 out of 81), whereas the percentage of patients with positive SARS-CoV-2 IgG levels (>31.5 BAU/ml) in patients who received the second dose only was 86.4% (70 out 81) *P* = 0.026. The percentage of participants who received the third (booster) dose and achieved a positive SARS-CoV-2-neutralizing antibody level was 100%, and the percentage of patients was 88.9% (72 out 81) of the participants who received the second dose only (*P* = 0.009). Finally, four patients had 0 neutralizing antibody levels after the second dose (see Figure 4).

## Discussion

In this study, we found that most patients with IBD on infliximab combination therapy had positive SARS-CoV-2 IgG and neutralizing antibody concentrations 40–45 weeks post BNT162b2 vaccination regardless of whether patients received a booster dose or not. However, SARS-CoV-2 IgG and neutralizing antibody concentrations were lower in patients who received two doses only compared to patients who received the third (booster) dose. Our study highlighted the importance of the booster dose in the IBD population.

A study by Levin et al. (20) found that 6 months after receiving the second dose of the BNT162b2 vaccine, the humoral response was substantially decreased among persons 65 years of age or older and persons with immunosuppression. Levin et al. also showed that SARS-CoV2 IgG and neutralizing antibody levels were lower in older men and participants with immunosuppression.

Another important finding of our study is that the percentage of participants who received the third (booster) dose and achieved positive SARS-CoV-2-neutralizing antibody level was significantly higher at 100%, relative to the percentage of patients who received the second dose only (88.9%). A study by Bergwerk et al. (21) recently reported a correlation between neutralizing antibody titers and infectivity. Similar to our study, Deepak et al. (22) found that 88.7% (118 of 133) of patients with chronic inflammatory diseases such as IBD and rheumatoid arthritis treated with immunosuppressive medications achieved positive anti-SARS-CoV-2 IgG levels after two doses of the BNT162b2 or mRNA-1273 vaccine; however, in many cases, antibody levels were lower than in immunocompetent participants. Another study looked at the SARS-CoV-2 neutralization antibody levels after the second-vaccine dose. Similarly, the study found that 85% of infliximab-treated patients and 86% vedolizumab-treated patients achieved positive neutralizing antibody levels (23). These studies suggested that although the specific threshold of antibody levels that can confer protection against breakthrough infection is still unclear, neutralizing antibodies may still be used to determine the efficacy and protection of vaccines.

The effect of TNF antagonists on serological responses of COVID-19 vaccination is becoming well documented in the literature. One study (15) recruited 362 patients with IBD treated with different immunosuppressive treatment regimens and 121 healthy controls. The authors found that patients treated with infliximab or tofacitinib had lower anti-SARS-CoV-2 spike protein antibody concentrations after two doses of vaccine than healthy controls. On the other hand, reductions in antibody responses were not observed in patients with IBD being treated with thiopurines, ustekinumab, or vedolizumab compared to control participants. Surprisingly, patients treated with infliximab experienced a 10-times reduction in anti-SARS-CoV-2 spike protein antibody concentrations relative to the control group. Similarly, another study by Shehab et al. recruited 116 patients with IBD receiving infliximab combination therapy. The authors concluded that in patients with IBD receiving infliximab combination therapy, SARS-CoV2 IgG, IgA, and neutralizing antibody levels were lower than the healthy participants (16). Finally, Melmed et al. (23) published a study that assessed antibody titers in adults with IBD who received mRNA SARS-CoV-2 vaccination. The study included 582 participants with IBD receiving different immunosuppressive therapies who have been vaccinated with either BNT162b2 or mRNA-1273 vaccines. The authors found that the mean SARS-CoV-2 antibody levels at 8 weeks were the highest among those treated with anti-integrin and anti-interleukin-12/23 and lowest among those treated with anti-TNFs combination therapy or corticosteroids; however, the study was not powered to assess differences across medication subgroups. In our study, we also noticed wide variability in the range of IgG and neutralizing antibodies, which could be attributed to age and comorbidity differences among patients. However, the range was narrow after the booster dose, which supports the recommendation for a third dose.

Comparing the efficacy of frequently administered vaccines, such as Hepatitis B Virus (HBV) and Influenza, in IBD patients can expand our understanding of viral vaccination interactions amid IBD therapies. Prat et al. reported considerably reduced seroprotective levels after HBV vaccinations (HBsAb ≥ 10 and 100 IU/L) among IBD patients receiving anti-TNF therapy (46.3%; *P* < 0.01) than those who did not. Furthermore, the report also mentioned that IBD patients receiving anti-TNF therapy combined with immunomodulators had worse outcomes, with 40.9% achieving levels adequate for protection (*P* < 0.001) (24). Similarly, Shirai et al. found that patients receiving anti-TNF therapies, such as infliximab, revealed decreased mean antibody titer after influenza vaccination. This was especially true in patients receiving infliximab combination therapy. Despite the overall reduction, patients still achieved sufficient levels of protection from a single influenza vaccine (25). These reports follow a comparable trend observed among COVID-19 vaccinated IBD patients. Like COVID-19 vaccines, HBV and Influenza vaccines among patients receiving anti-TNF therapy, especially infliximab (with or without combination therapy), exhibited lower seroprotective levels.

Our results build on growing literature confirming that most patients with IBD can mount humoral responses after the second dose of SARS-CoV-2 vaccination, with a small proportion generating poor or no response, which justifies current recommendations for this population to receive a booster dose of BNT162b2 vaccine. In addition, our pre-defined inclusion and exclusion criteria lower the risk of confounding bias, and patients were equally distributed and matched in terms of demographic characteristics such as age, sex, and BMI.

This study has some limitations. We cannot rule out that some of the included patients might have had silent COVID-19 at the time of recruitment, such as by doing SARS-CoV-2 N protein-specific IgG tests, which could have provoked a serological response (26). However, we did PCR testing before each vaccine dose and excluded any patients with current or previous symptoms of COVID-19. In addition, we only assessed positive IgG and neutralizing antibodies. However, we did not investigate cellular immunity, B-, and T-cell responses, which may also play a role in vaccine efficacy. We assessed infliximab with azathioprine or 6-mercaptopurine only. Further studies are needed to investigate the effect of other immunomodulators. Finally, we did not compare our data to a healthy control group.

## Conclusion

Most patients with IBD on infliximab combination therapy had positive SARS-CoV-2 IgG and neutralizing antibody concentrations 40–45 weeks post BNT162b2 vaccination. However, SARS-CoV-2 IgG and neutralizing antibody concentrations were lower in patients who received two doses relative to patients who received the third (booster) dose. A longer follow-up study is needed to evaluate the decay in antibodies over time.

## Data Availability Statement

The datasets presented in this article are not readily available because local legal restrictions. Requests to access the datasets should be directed to mkh@moh.edu.kw.

## Ethics Statement

The studies involving human participants were reviewed and approved by the Ethical Review Board of Dasman Institute Protocol # RA HM-2021-008 as per the updated guidelines of the Declaration of Helsinki (64th WMA General Assembly, Fortaleza, Brazil, October 2013) and of the US Federal Policy for the Protection of Human Subjects. The study was also approved by the Ministry of Health of Kuwait (reference: 3799, protocol number 1729/2021). Subsequently, patient informed written consent was obtained before inclusion in the study. The patients/participants provided their written informed consent to participate in this study.

## Author Contributions

MS: study concept and design, acquisition of data, analysis and interpretation of data, drafting of the manuscript, critical revision of the manuscript for important intellectual content, and submission of the manuscript. FA, AAlf, AAls, and UA: acquisition of data and drafting of the manuscript. PC and IA: statistical analysis. TT and AC: interpretation of data. AD, AAlb, and HA: data collection and supervision. MA-F, JA, and FA-M: critical revision of the manuscript for important intellectual content and study supervision. All authors contributed to the article and approved the submitted version.

## Funding

This study was funded by the Kuwait Foundation for the Advancement of Sciences (KFAS) grant (RA HM-2021-008).

## Conflict of Interest

The authors declare that the research was conducted in the absence of any commercial or financial relationships that could be construed as a potential conflict of interest.

## Publisher's Note

All claims expressed in this article are solely those of the authors and do not necessarily represent those of their affiliated organizations, or those of the publisher, the editors and the reviewers. Any product that may be evaluated in this article, or claim that may be made by its manufacturer, is not guaranteed or endorsed by the publisher.
